# Aging envisage imbalance of the periodontium: A keystone in oral disease and systemic health

**DOI:** 10.3389/fimmu.2022.1044334

**Published:** 2022-10-20

**Authors:** Verónica Villalobos, Mauricio Garrido, Antonia Reyes, Christian Fernández, Catalina Diaz, Vicente A. Torres, Pablo A. González, Mónica Cáceres

**Affiliations:** ^1^ Millennium Institute on Immunology and Immunotherapy, Santiago, Chile; ^2^ Program of Cellular and Molecular Biology, Institute of Biomedical Sciences, Faculty of Medicine, Universidad de Chile, Santiago, Chile; ^3^ Department of Conservative Dentistry, Faculty of Dentistry, Universidad de Chile, Santiago, Chile; ^4^ Departamento de Genética Molecular y Microbiología, Facultad de Ciencias Biológicas, Pontificia Universidad Católica de Chile, Santiago, Chile; ^5^ Institute for Research in Dental Sciences, Faculty of Dentistry, Universidad de Chile, Santiago, Chile; ^6^ Advanced Center for Chronic Diseases (ACCDiS), Universidad de Chile, Santiago, Chile; ^7^ Millennium Nucleus of Ion Channels-Associated Diseases (MiNICAD), Universidad de Chile, Santiago, Chile

**Keywords:** aging, gingival fibroblast, macrophage, histatin, herpes simplex virus, *Porphyromonas gingivalis*

## Abstract

Aging is a gradual and progressive deterioration of integrity across multiple organ systems that negatively affects gingival wound healing. The cellular responses associated with wound healing, such as collagen synthesis, cell migration, proliferation, and collagen contraction, have been shown to be lower in gingival fibroblasts (the most abundant cells from the connective gingival tissue) in aged donors than young donors. Cellular senescence is one of the hallmarks of aging, which is characterized by the acquisition of a senescence-associated secretory phenotype that is characterized by the release of pro-inflammatory cytokines, chemokines, growth factors, and proteases which have been implicated in the recruitment of immune cells such as neutrophils, T cells and monocytes. Moreover, during aging, macrophages show altered acquisition of functional phenotypes in response to the tissue microenvironment. Thus, inflammatory and resolution macrophage-mediated processes are impaired, impacting the progression of periodontal disease. Interestingly, salivary antimicrobial peptides, such as histatins, which are involved in various functions, such as antifungal, bactericidal, enamel-protecting, angiogenesis, and re-epithelization, have been shown to fluctuate with aging. Several studies have associated the presence of *Porphyromonas gingivalis*, a key pathogen related to periodontitis and apical periodontitis, with the progression of Alzheimer’s disease, as well as gut, esophageal, and gastric cancers. Moreover, herpes simplex virus types 1 and 2 have been associated with the severity of periodontal disease, cardiovascular complications, and nervous system-related pathologies. This review encompasses the effects of aging on periodontal tissues, how *P. gingivalis* and HSV infections could favor periodontitis and their relationship with other pathologies.

## Periodontium and aging

Periodontium is a tissue that supports the teeth and protects against oral pathogens. Anatomical and functional changes in periodontal tissues have been associated with aging, including thinning of the epithelium and diminished keratinization, whereas cementum increases in width. Consequently, periodontal health decreases with aging (1).

**Figure 1:**
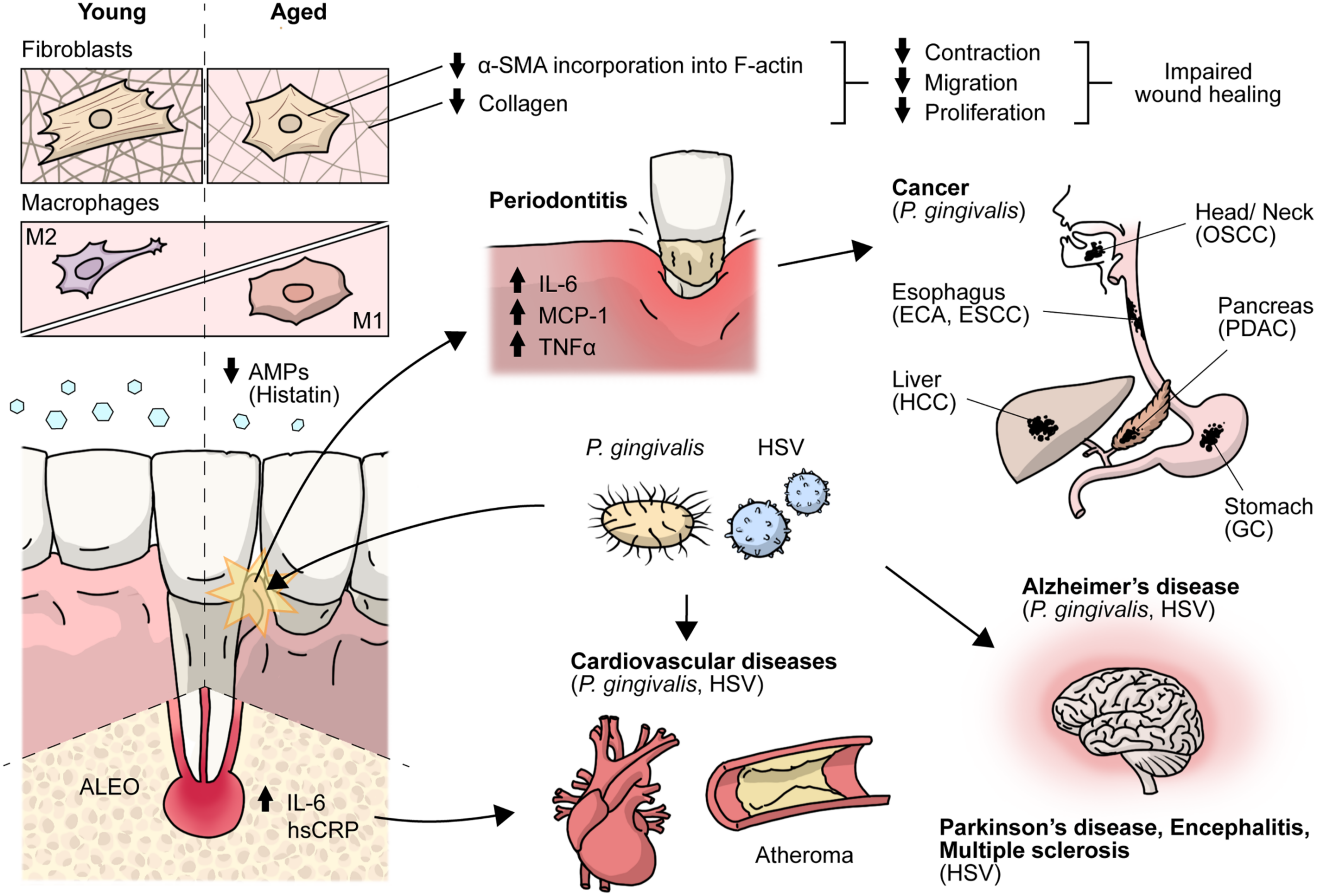
Effects of aging on the periodontium and antimicrobial peptides. Crosstalk between HSV and *P.gingivalis* with cancer, encephalitis, multiple sclerosis, cardiovascular, Parkinson’s, Alzheimer’s and periodontal diseases. During aging there is an impaired gingival wound healing. Aged gingival fibroblasts show decreased cell migration, proliferation and contraction, and lower α-SMA is incorporated into actin stress fibers and aged macrophages show altered acquisition of functional phenotypes, HSV and *P*.*gingivalis* have been associated with periodontal, cardiovascular and Alzheimer’s diseases. AMPs, antimicrobial peptides; ALEO, apical lesions of endodontic origin; hsCRP, high-sensitive C-reactive protein; α-SMA, alpha-smooth muscle actin; OSCC, oral squamous cell carcinoma; ESCC, esophageal squamous cell carcinoma; PDAC, pancreatic ductal adenocarcinoma; HCC, hepatocellular carcinoma; GC, gastric cancer.

**Figure 2:**
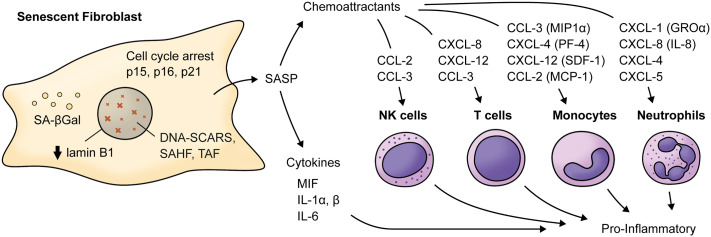
Main cytokines and chemokines elevated in the Senescence-Associated Secretory Phenotype by senescent fibroblasts. Senescent fibroblasts are characterized by cell-cycle arrest mainly related to increased levels of cyclin-dependent kinases inhibitors (p15^INK4b^, p16^INK4a^, p21^CIP1^), decreased levels of lamin B1 and the presence of a SASP characterized by increased levels of cytokines and chemokine such as IL-6, MIF, IL-1α and β which acts as pro-inflammatory cytokines. For example, chemokines such as CXCL-1, CXCL-8, CXCL-5, and CXCL-4 can be chemoattractants for neutrophils. CCL-3, CCL-4, CXCL-12, and CCL-2 can be chemoattractants for monocytes, and IL-8, CXCL-12, and CCL-3 can act as a chemoattractant for T cells. Notably, CCL-2 and CCL-3 can also attract NK cells. MIF, macrophage migration inhibitory factor; MIP1α, macrophage inflammatory protein 1-alpha; MCP-1, monocyte chemoattractant protein 1; CXCL-5, C-X-C motif chemokine 5; PF-4, platelet factor 4; SDF-1, stromal cell-derived factor 1; SAHF, Senescence-associated heterochromatin foci; TAF, Telomere-associated DDR foci; DNA-SCARS, DNA segments with chromatin alterations reinforcing senescence. SA-βGal, Senescence-associated beta galactosidase; GROα, growth-regulated alpha protein.

Aging is a biological process characterized by decreased cell function that negatively affects gingival wound healing (2). Different cellular responses associated with wound healing, such as cell migration, proliferation, and collagen contraction, have been found to be lower in gingival fibroblasts (GF) derived from aged donors than in those derived from young donors (2). Accordingly, collagen production decreases by more than five-fold depending on the age of the donor (3), while old GF show increased rates of collagen phagocytosis and augmented DNA methylation in the collagen alpha-1 gene, which is followed by a reduction in mRNA levels and collagen type I synthesis (3). Interestingly, TGF-β1 stimulation increased the α-SMA levels in both young and old fibroblasts. However, α-SMA is incorporated in actin stress fibers in young fibroblasts but not in old fibroblasts (2) (**Figure 1**). One of the proteins that increase its expression during aging in human GF is TMPRSS11a (4), a type II serine protease that induces cellular senescence, a process characterized by stable cell cycle arrest, macromolecular damage induced by cellular impairment, such as DNA damage (4), telomere shortening or dysfunction, epigenetic changes, oncogene activation or loss of tumor suppressor functions, and organelle damage and with the acquisition of a senescence-associated secretory phenotype (SASP) (5). The SASP is characterized by the release of components that directly or indirectly promote inflammation such as pro-inflammatory cytokines, chemokines, growth factors, and proteases (5). This phenotype has been implicated in the recruitment of immune cells (**Figure 2**), impacting the local oral mucosal microenvironment and affecting cellular function in neighboring cells (6).

A study from our group showed that the exposure of young GF to blood serum from middle-aged (30-48 years old) and aged individuals (over 50 years old) increased cellular senescence. Specifically, that study showed that blood serum samples obtained from middle-aged and aged individuals were characterized by an increase in MCP-1 (CCL2) and TNFα levels compared to those in young individuals (6). Interestingly, one longitudinal study quantified physiological deterioration across multiple organ systems with chronological aging, using a 38-year-old, showing that gum health (combined with attachment loss) has a Z score similar to other biomarkers of aging, such as HDL cholesterol and telomeric length in leukocytes (7). Interestingly, the authors used the age of 38-year-old because individuals who were aging more rapidly were less physically able, showed cognitive decline and brain aging and looked older, so they can identify causes of aging and evaluate rejuvenation therapies.

During aging, there is a loss of periodontal attachment and alveolar bone, but these changes do not have a major clinical impact. However, in aged individuals, there is an increased pro-inflammatory state that induces increased susceptibility to autoimmune, inflammatory, or infectious diseases, including periodontitis (8). Furthermore, in the gingival tissues of aged subjects, differences associated with the innate immune system, such as higher neutrophil infiltration in gingiva from old mice (18 months old) compared to young mice, where the reduced expression of Del-1 in the gingiva of old mice was associated with higher neutrophil infiltration relative to young mice (9). The innate immune system plays a critical role in maintaining a symbiotic relationship with the oral microbiome, were observed (1). Thus, alterations in host oral mucosal immunity arise during aging that giving way to the establishment of oral dysbiosis that allows tissue colonization by bacteria such as *Porphyromonas gingivalis (P. gingivalis)* (1), which increases susceptibility to the development of periodontitis or latent viral infections.

## Antimicrobial peptides

Antimicrobial peptides (AMPs) are central players in innate immunity, which depict additional activities beyond their canonical antimicrobial roles, thereby contributing to maintaining tissue homeostasis (10). Within the group of salivary AMPs, histatins stand out, as their concentrations fluctuate in early and middle ages and further decrease in the elderly (11) (**Figure 1**). Histatins are histidine-rich proteins that elicit a variety of functions, including antifungal, bactericidal, and enamel-protective activities (12). From this family of proteins, histatin-1 and histatin-2 have been extensively studied, as they have been found to maintain oral mucosal homeostasis and promote wound healing by stimulating both tissue re-epithelialization (13) and angiogenesis (14). Particularly, histatin-1 was identified as a potent pro-angiogenic factor that stimulates endothelial cell adhesion and migration, as well as vascular morphogenesis *in vitro* and *in vivo* (14), which are thought to contribute to the high efficiency of epithelial repair in the oral cavity. In addition to its effects on wound healing, histatin-1 was also reported to contribute to maintaining periodontal tissue homeostasis, because it restores cell migration in periodontal ligament fibroblasts challenged with nicotine (14). In support of the notion that histatin-1 contributes to oral fibroblast function, this peptide induced myofibroblast differentiation and migration in a non-oral model, thereby contributing to skin wound healing (15).

The spectrum of cell types that respond to histatin-1 has broadened in recent years, as this peptide was shown to induce osteogenic differentiation and stimulate mineralization in pre-osteoblasts and mesenchymal cells derived from the dental pulp and apical papilla (16). Collectively, these findings open new avenues to explore the physiological relevance of the effects of histatin-1 in different cell types residing within the oral cavity. In addition, this scenario provides an interesting opportunity to explore new therapeutic possibilities using this peptide as a co-adjuvant in regenerative therapies. This is relevant in the context of the reported variations in this and other histatins during aging (11).

## Periodontal disease

Periodontitis is known to increase in both incidence and severity across a large proportion of the human population with aging (1) and has been associated with other age-related diseases, such as cancer, Alzheimer’s disease (AD), and atherosclerosis (17–19). The etiology of periodontal disease has been commonly associated with bacterial infection solely. However, it has been reported that a severe disease also occurs in the absence of a large bacterial load, indicating an excessive immune response of the host inflammatory cytokine-mediated to subgingival pathogens, where IL-1α plays a major role in periodontal damage (20). Alterations in the components of the periodontium that disrupt its barrier functions can lead to an increase in opportunistic pathogens, causing the development of diseases at both local and systemic levels. It is important to note that the oral microbiome can comprise bacteria, protozoans, fungi, numerous viruses, and archaea; in this review, we focus on discussing *P. gingivalis)* and herpes simplex virus type 1 and 2 (HSV-1 and HSV-2, respectively).

## *P. gingivalis* in supportive periodontal tissue diseases and systemic implications

*P. gingivalis* is a gram-negative anaerobic bacillus part of a cluster of oral microorganisms consistently found in severe forms of periodontal disease, classically described as “red complex”: *P. gingivalis*, *Tanerella Forsythia*, and *Treponema denticola* (17). *P. gingivalis* plays a central role in the etiology and progression of periodontal disease because of the wide range of virulence factors that are associated with the induction of a pro-inflammatory environment in the oral mucosa, leading to the destruction of periodontal tissue, diminishing its barrier function and immunological homeostasis, and hence responses against noxa (8). Inflammation favors the growth of the dysbiotic microbial community. Nevertheless, its disruptive effects on oral mucosal immunity transcend the inflammatory balance, as it also plays a role in mucosal senescence induction (21). Direct cellular invasion by this bacterium has been shown to induce immune senescence in dendritic cells, and paracrine signals amplify senescence through the secretion of inflammatory exosomes (21).

In addition, *P. gingivalis* can translocate from the oral cavity to the bloodstream and colonize distant organs, invading and surviving within dendritic cells and monocytes/macrophages (19, 21). In this context, *P. gingivalis* was shown to play a pathogenic role in systemic diseases, such as vascular atheroma, where it is located in atherosclerotic plaques (18). In addition, periodontal disease has been associated with an increased risk of cancers (**Figure 1**), such as pancreatic cancer, via a mechanism that relies on *P. gingivalis*, as it was found in pancreatic cancer cells and has been shown to exert direct pro-tumoral effects on enhanced pancreatic tumor cell proliferation through its ability to survive intracellularly (22). However, the conclusive mechanism of invasion in atheroma plaques and pancreatic adenocarcinoma is not completely understood.

Although *P. gingivalis* has been associated with local inflammation and oral cancer risk, recent evidence suggests that the consequences of oral mucosal imbalance might extend beyond local malignancies. Oral dysbiosis has been shown to increase the risk of oral squamous cell carcinoma (OSCC), and bacterial communities displaying enrichment of genes associated with cell motility and pro-inflammatory processes, such as bacterial chemotaxis and flagella assembly, are significantly increased in OSCC patients (23). *P. gingivalis* is also associated with other gut cancers, such as esophageal cancer, gastric cancer, and hepatocarcinoma (17).

In contrast, *P. gingivalis*-derived gingipains and LPS, as well as their DNA, have been found in the brains of individuals with AD, suggesting transneuronal dissemination of this bacterium from the oral cavity (19). Interestingly, *P. gingivalis* is also present in individuals with AD but without established dementia, suggesting that brain infections are an early event that plays a role in AD pathogenesis (19) (**Figure 1**). Mechanisms underlying neurodegeneration are related to inflammation mediated by *P. gingivalis* virulence factors, including LPS, gingipains, cathepsin B, and tau, among others (24), which lead to the accumulation and production of the amyloid plaque component Aβ in the brain and tau-related pathology, as a result of gingipain proteolysis (19), although clinical studies correlating these data are still incipient.

Another prevalent oral disease worldwide that affects periapical supportive tissues and may cause loss of the affected tooth is apical periodontitis (25). It usually presents as an asymptomatic disease that is radiographically detected as an osteolytic area. The etiological factor is a predominantly anaerobic biofilm that triggers an immune-inflammatory response by the host (26), where macrophages play an important role in the hallmark of apical lesions of endodontic origin (ALEOs), such as root resorption. There is evidence that these ALEOs depict a high percentage of extraradicular infections, specifically *P. gingivalis* and *Porphyromonas endodontalis*, thus challenging the notion that microorganisms are confined only to the root canal system (27). Furthermore, bacterial DNA has been detected at significantly higher levels in peripheral blood mononuclear cells taken from individuals with apical periodontitis than in healthy controls, suggesting that there might be bacterial DNA translocation from the ALEO onto the systemic circulation, reaching other tissues, such as endothelial cells of blood vessels (27). It has also been shown that *Porphyromonas* spp. induces autoimmune responses that may contribute to cardiovascular diseases; specifically, *P. gingivalis*, which can invade endothelial cells (28).

Serum levels of high-sensitivity C-reactive protein (hsCRP), used as a biomarker for a cardiovascular event, are higher in young individuals with AP than in healthy controls, as well as IL-6, matrix metalloproteinase 8 (MMP-8), and soluble E-selectin, which are implicated in atherogenesis (29) (**Figure 1**). There is even an association of ALEOs with hsCRP levels > of 3 mg/mL, supporting a mechanistic link between this prevalent disease and cardiovascular risk in young individuals (30).

## Role of macrophages during aging

Macrophages are phagocytic cells that form the first line of defense against pathogens. These plastic cells can polarize from classically activated M1 macrophages to alternatively activated M2 macrophages *in vitro*. However, the polarization state of M1 and M2 macrophages *in vivo* corresponds to a continuum of intermediate phenotypes that can switch from one phenotype to another in response to the cytokine milieu in each tissue microenvironment, whereas an inadequate balance between the polarization states can lead to the development of chronic inflammation and disease (31).

During aging, altered acquisition of functional phenotypes in response to the tissue microenvironment has been reported in splenic macrophages (32) and bone-marrow-derived macrophages (BMMs) (33) obtained from aged mice compared to young mice. The mRNA levels of pro-inflammatory cytokines (i.e. IL-6, TNFα, iNOS, and IL-1β) from aged splenic macrophages were decreased after LPS from *Escherichia coli* (*E. coli*) or TNFα/IFN-γ stimulation, as compared with cells derived from younger animals (32). These results are concordant with a reduced amount of pro-inflammatory cytokines, including IL-12, and increased IL-10, produced by splenic macrophages, as previously reported (34). Conversely, aged mouse BMMs from mice showed higher levels of the TNFα transcript and protein secretion after IFN-γ and *E. coli*-LPS stimulation, respectively (33). However, BMMs challenged with *P. gingivalis*, a common periodontal pathogen, show attenuated levels of cytokines and chemokines, including TNFα, IL-6, IL-10, and nitric oxide (NO) (35). Furthermore, aged macrophages showed lower levels of TLR4/MD-2, although no changes were observed in their surface density (34).

Macrophages play a role in the innate host response in periodontitis, particularly in oral tissue. Clark *et al.* (36) reported age-related changes in macrophages associated with a pro-inflammatory and M1-like phenotype (**Figure 1**), as well as improper polarization, which might be associated with the impaired inflammatory regulatory activity of macrophages in response to microbial plaque and periodontal disease resolution. Likewise, decreased NO production and changes in the expression levels of toll-like receptors (TLRs), together with defects in the signaling pathways in aged macrophages (37, 38), could be related to increased susceptibility to infections in the elderly and chronic disease development such as periodontitis.

## Herpes simplex virus infection in periodontal disease and systemic implications

HSV-1 and HSV-2 are two highly prevalent viruses in the human population. It is estimated that approximately 66.6% of individuals worldwide are infected with HSV-1 and 13.2% with HSV-2 (39). Both viruses have been reported to modulate the host´s innate and adaptive immune responses (40, 41). HSVs cause lifelong infections with a wide range of clinical manifestations, from mild to life-threatening. A common primary infection-related clinical manifestation in children is herpetic gingivostomatitis, which consists of oral lesions in the buccal and gingival mucosae as well as the tongue, and is usually self-contained (39).

Importantly, HSV-1 and HSV-2 have been associated with the severity of periodontal diseases, such as chronic inflammatory conditions that affect the supporting tissue of the teeth (42). Notably, HSV DNA has been found in subgingival plaque samples associated with higher clinical attachment loss (≥4 mm) and the presence of bleeding on probing (BOP) (43). Moreover, it has been estimated that 63% of sites with aggressive periodontitis and 45% of sites with chronic periodontitis contained HSV-1 copy counts (44)

The severity of periodontal diseases related to HSV infections may be explained by the fact that herpes simplex viruses infect periodontal tissues (**Figure 1**), which induces local immune responses and may also serve as cofactors for bacterial virulence determinants in periodontal diseases (43). Interestingly, HSV-1 and HSV-2 DNA have been detected *via* nested PCR in T cells and monocytes/macrophages in gingival cells obtained from adult individuals with periodontitis (45). Furthermore, it was found that downregulation of periodontal T cell function caused by HSV infection may increase the risk of destructive periodontal disease, as T cells have been suggested to have a protective role in periodontal disease (45).

Importantly, the combination of bacteria associated with periodontitis and HSV infections could lead to an increased likelihood of cardiovascular complications in infected individuals (46). Indeed, HSV-1 and HSV-2 have been associated with several cardiac diseases (**Figure 1**), such as atherosclerosis, and are considered factors that increase the risk of this disease (47). Noteworthy, two mechanisms have been proposed to explain the association between periodontitis and cardiovascular diseases such as atherosclerosis. Either periodontal pathogens directly invade the bloodstream or indirectly increase the systemic levels of inflammatory mediators (47). Importantly, it has been suggested that improving one of these conditions may positively affect the other diseases (47). However, this potential association requires further investigation.

HSV infections may cause nervous system-related pathologies, such as herpetic encephalitis, meningitis, and Mollaret’s syndrome, and have been associated with several neurodegenerative diseases, such as Parkinson’s disease, multiple sclerosis (MS), and AD (48) (**Figure 1**). Moreover, several studies have associated HSVs infections with neuronal aging, which, in turn, could lead to AD. For instance, it has been shown both *in vitro* and *in vivo* models that HSV-1 infection induces a significant increase in the levels of histone modifications, such as H4 lysine (K) 16 acetylation (ac) (H4K16ac) and histone-modifying factors, such as Sin3 and histone deacetylases (HDAC1), suggesting that neuronal responses to virus latency and reactivation upregulate these aging markers (49). Additionally, upregulation of the histone regulator A (HIRA) during viral latency and its different localization in cortical neurons in HSV-1-infected mouse brains have also been reported (49). Importantly, HIRA is also a key player in aging (49). Thus, HSVs infections are also linked to aging of the nervous system, which is directly related to the development of AD.

Interestingly, the frequency and magnitude of herpesvirus reactivation have been described to be affected by aging. This observation is supported by the detection of increased CD8+ T cells specific for this virus in aged subjects. However, herpesviruses can also reactivate as a result of stress. Further studies are needed to understand the effects of herpesvirus reactivation and its role in healthy aging (50).

## Conclusions

There are changes at both immune and non-immune cells of the oral mucosa during aging, that lead to a destabilization of the tissue homeostasis and impaired wound healing. Also, these aging-associated changes coupled with the decrease in AMPs create a permissive environment in the periodontium for both pathogen colonization and reactivation of pre-existing pathogens such as HSV, which may influence the development and severity of oral diseases, such as periodontitis. Interestingly, pathogens involved in the development of this disease, such as *P.gingivalis*, are also involved in the development of systemic diseases including various cancers and Alzheimer’s disease. Also, HSV infections at other sites than the oral cavity may nevertheless relate to periodontitis and central nervous system diseases, given the potential of this condition to cause systemic diseases. Given this scenario, further studies on these eventual associations may shed light on previously unrecognized relations between cell and tissue aging and oral diseases as well as new insight into their consequences at the systemic level.

## Author contributions

VV and MC were involved in designing the concept of the review and oversight. AR and PG were involved in the HSV section and revisions of the manuscript. VAT was involved in the AMP section and revisions of the manuscript. CF and CD were involved in the Periodontal section. VV was involved in the macrophages and *P.gingivalis* sections and drafted the manuscript. MG was involved in the apical periodontal disease section and revisions of the manuscript and MC was involved in the aging section and drafted the manuscript. All authors contributed to the article and approved the submitted version.

## Funding

Millennium Institute on Immunology and Immunotherapy (ICN09_016/ICN 2021_045). Financiado por la Vicerrectoría de Investigación y Desarrollo (VID) de la Universidad de Chile, proyecto ENL05/22.

## Acknowledgments

We are grateful to Mr. Diego Morales for drawing both figures. Agencia Nacional de Investigación y Desarrollo (ANID) - Millennium Science Initiative Program - ICN09_016/ICN 2021_045: Millennium Institute on Immunology and Immunotherapy (ICN09_016/ICN 2021_045; former P09/016-F). Financiado por la Vicerrectoría de Investigación y Desarrollo (VID) de la Universidad de Chile, proyecto ENL05/22. Beca doctorado ANID 21210960 (CF), 21211818 (VV), 21210509 (MG), 21210570 (CD).

## Conflict of interest

The authors declare that the research was conducted in the absence of any commercial or financial relationships that could be construed as a potential conflict of interest.

## Publisher’s note

All claims expressed in this article are solely those of the authors and do not necessarily represent those of their affiliated organizations, or those of the publisher, the editors and the reviewers. Any product that may be evaluated in this article, or claim that may be made by its manufacturer, is not guaranteed or endorsed by the publisher.
